# Impact of dietary zinc oxide nanoparticles on selected serum biomarkers, lipid peroxidation and tissue gene expression of antioxidant enzymes and cytokines in Japanese quail

**DOI:** 10.1186/s12917-020-02482-5

**Published:** 2020-09-23

**Authors:** Sabry Mohamed El-Bahr, Saad Shousha, Ibrahim Albokhadaim, Ahmed Shehab, Wassem Khattab, Omar Ahmed-Farid, Osama El-Garhy, Abdelrahman Abdelgawad, Mehrez El-Naggar, Mahmoud Moustafa, Omnia Badr, Mohammad Shathele

**Affiliations:** 1 Department of Biomedical Sciences, College of Veterinary Medicine, King Faisal University, P.O. Box 400, Al- Ahsa, Saudi Arabia; 2 Department of Biochemistry, Faculty of Veterinary Medicine, Alexandria University, Alexandria, Egypt; 3 Department of Physiology, Faculty of Veterinary Medicine, Benha University, Benha, Egypt; 4 Department of Nutrition and Clinical Nutrition, Faculty of Veterinary Medicine, Benha University, Qalioubia, Moshtohor, Benha, Egypt; 5 Department of Physiology, National Organization for Drug Control and Research, Giza, Egypt; 6 Department of Animal Production, Faculty of Agriculture, Benha University, Qalioubia, Moshtohor, Benha, Egypt; 7 Textile Research Division, National Research Centre, Dokki, Egypt; 8 Department of Genetics and Genetic Engineering, Faculty of Agriculture, Benha University, Benha, Egypt; 9 Department of Microbiology, College of Veterinary Medicine, King Faisal University, Al-Ahsa, Saudi Arabia

**Keywords:** Japanese quails, Zinc oxide nanoparticles, Gene expression, Antioxidants

## Abstract

**Background:**

The use of zinc oxide in the form of nanoparticles (ZnO-NPs) is of great benefit due to its potent effectiveness and higher bioavailability compared to zinc oxide. This study aimed to investigate the impact of dietary inclusion of different doses of ZnO-NPs on selected serum biomarkers, lipid peroxidation and tissue gene expression of antioxidant enzymes and cytokines in Japanese quail. Eighty Japanese quails (*Coturnix japonica*) (45 days old) were randomly divided into four groups (20 birds for each) with 4 replicates (5 birds each). Birds in the first group were fed a basal diet alone and served as a control (C). Birds in groups 2–4 were fed the basal diet supplemented with ZnO-NPs at doses of 15 mg/kg, 30 mg/kg and 60 mg/kg for a period of 60 days. At the end of the experiment, all birds were sacrificed to collect blood in a plain vacutainer, whereas liver and brain tissues were stored frozen at -80 °C. The obtained sera were used for the analysis of selected biochemical parameters, whereas tissue homogenates were used for the estimation of zinc, oxidative stress biomarkers and gene expression of selected antioxidant enzymes and cytokines.

**Results:**

ZnO-NPs (30 and 60 mg/kg) induced a significant decrease in serum triacylglycerol (TAG) compared to the control. ZnO-NPs did not affect the activities of serum alanine aminotransferase (ALT), aspartate aminotransferase (AST), total protein, albumin, globulin and tissue zinc concentrations but reduced the malondialdehyde (MDA) levels compared to the control. The liver retained a higher zinc concentration than that of brain tissue. In a dose-dependent manner, ZnO-NPs upregulated the mRNA levels of antioxidant enzymes (superoxide dismutase: SOD1; catalase: CAT; glutathione peroxidase-1: GPX 1) and pro-inflammatory cytokines (interferon α: IFN-α; interleukin 6: IL-6) in liver and brain tissues.

**Conclusion:**

The current study suggests the inclusion of ZnO-NPs, particularly 60 mg/kg, in the diet of Japanese quails to improve antioxidant and immune status.

## Background

Zinc is an essential trace element for poultry. It improves the growth performance, skeletal muscle development and immunity of broiler chickens [1]. Due to the low bioavailability of zinc, it is important to ensure the requirements of animals by increasing the concentration of inorganic Zn approximately 20- to 30-fold above the optimum requirements [2]. However, this scenario is costly, might create a problem with higher zinc residues in chicken manure [3] and can reduce the digestibility of other minor elements, such as copper, iron, and cadmium [4]. Therefore, the improvement in Zn bioavailability is a major prerequisite for the solution to this problem. Recently, there has been increasing interest in substituting bulk inorganic minor elements with nanoparticles. These substitutions increase the surface reactive area, bioavailability, and absorbability, as well as cover the needs of the animals accordingly [5, 6]. In this context, zinc oxide nanoparticles (ZnO-NPs) are considered suitable candidates as feed additives [7]. The addition of ZnO-NPs to poultry diets induced significant decreases in serum total cholesterol (TC) and triacylglycerol (TAG) [8]. This effect may be linked to the role of zinc in enhancing fat metabolism or reducing the absorption of dietary lipids [8]. Dietary ZnO-NPs reduced the malondialdehyde (MDA) concentration and increased superoxide dismutase (SOD) and catalase (CAT) activities in chickens [8–10]. Dietary ZnO-NPs (500 µg/litre) increased the expression of the SOD, CAT and glutathione peroxidase (GPX) genes in tilapia [11]. This explains role of zinc in enhancing the antioxidant status in birds [8]. ZnO-NP supplementation potentiated the mRNA expression of IL-1β, IL-10, and TNF-α [12]. However, studies regarding the effect of ZnO-NPs in Japanese quails are insufficient and require further investigation. Therefore, the current study aimed to investigate the impact of dietary inclusion of different doses of ZnO-NPs on selected serum biomarkers, lipid peroxidation and tissue gene expression of antioxidant enzymes and cytokines in Japanese quail.

## Results

### Characterization of ZnO-NPs

**Fig. 1:**
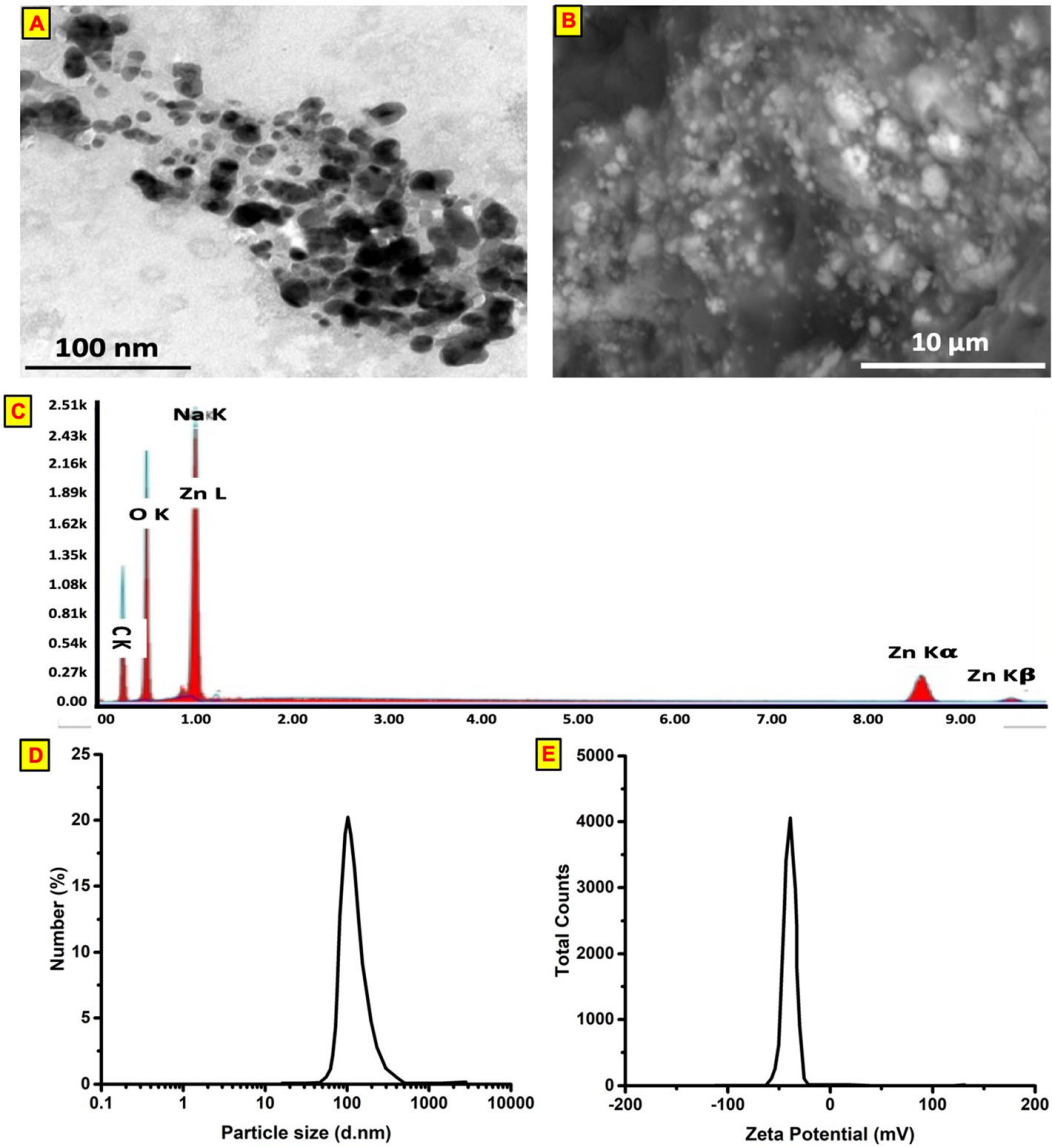
Characterization of zinc oxide nanoparticles. **a** a high-resolution transmission electron microscope (HRTEM) images of ZnO-NPs where its size is between 40 nm. **b** The field emission scanning electron microscopy (FESEM) images of ZnO-NPs where its particle size was approximately 60–95 nm. **c** Energy dispersive X-ray spectra (EDS) of ZnO-NPs. **d** average size distribution and **e** surface charge of prepared ZnO-NPs by using dynamic light scattering technique via Zetasizer instrument

High-resolution transmission electron microscopy (HRTEM) images of ZnO-NPs indicate the spherical shape of the obtained nanocrystals of ZnO and a particle size of 40 nm (Fig. 1a). The field emission scanning electron microscopy (FESEM) images of ZnO-NPs (Fig. 1b) showed that ZnO-NPs are identical, spherical with a smooth surface and approximately 60–95 nm in size. Peaks of elemental zinc are present in different oxidation states (Fig. 1c). The average size distribution and the surface charge, as indicated by a Zetasizer, were determined to be approximately 130 nm, and the apparent Zeta potential was approximately − 42 mV (Fig. 1d and e).

### Effect of dietary ZnO-NP supplementation on selected serum biochemical parameters

The activities of liver enzymes (AST and ALT) and the concentrations of total proteins, albumin and globulin remained unchanged in the serum of Japanese quail fed ZnO-NPs compared to the control (Table 1). Total cholesterol (TC) decreased significantly (*p* < 0.05) in the serum of Japanese quail fed the highest dose of ZnO-NPs (60 mg/kg diet; group 4) compared to the other groups, including the control (Table 1). In addition, serum TAG decreased significantly in birds fed 30 mg/kg diet (group 3) and 60 mg/kg diet (group 4) of ZnO-NPs compared to the control (Table 1). The serum TAG of birds fed lowest dose of ZnO-NPs (group 2; 15 mg/kg) did not change significantly compared to the control group (Table 1). The zinc concentration was increased significantly in the serum of Japanese quails fed the highest dose of ZnO-NPs (60 mg/kg; group 4) compared to the other groups, including the control (Table 1).

**Table 1:** Effect of dietary ZnO-NPs supplementation on serum selected biochemical parameters in Japanese quails

Parameters	Dietary groups				SEM	*P* values
	Group 1	Group 2	Group 3	Group 4		
AST (U/L)	16.02	17.07	16.35	17.62	0.48	0.695
ALT (U/L)	38.25	35.05	34.42	32.72	0.93	0.206
Total Proteins (mmol/L)	0.459	0.487	0.456	0.469	0.15	0.713
Albumin (mmol/L)	0.173	0.171	0.185	0.186	0.20	0.618
Globulin (mmol/L)	0.241	0.285	0.226	0.240	0.50	0.589
TC (mmol/L)	5.28^a^	5.00^a^	4.90^a^	4.50^b^	0.24	0.014
TAG (mmol/L)	5.31^a^	5.03^a^	4.66^b^	4.54^b^	0.29	0.006
Zinc (µmol/L)	10.80^b^	12.39^b^	13.13^b^	19.70^a^	0.65	0.004

^a−c^ Means within a row not sharing a common superscript differ significantly with corresponding *p* value

Group 1: control; Group 2: ZnO-NPs 15 mg/kg diet; Group 3: ZnO-NPs 30 mg/kg diet; Group 4: ZnO-NPs 60 mg/kg diet

*Abbreviations*: *ZnO-NPs* zinc oxide Nano particles, *SEM* Standard error of mean, *ALT* alanine aminotransferase, *AST* aspartate aminotransferase, *TC* total cholesterol, *TAG* triacylglycerol

### Effect of dietary ZnO-NP supplementation on lipid peroxidation and oxidative stress biomarkers in liver and brain tissues

The data summarized in Table 2 indicated that all studied doses of ZnO-NPs induced a significant reduction (*p* < 0.05) in MDA concentrations in liver and brain tissues of Japanese quails compared to the control. Furthermore, all studied doses of ZnO-NPs induced significant increases (*p* < 0.0001 and *p* < 0.01) in NO concentrations in the liver and brain tissues of Japanese quails compared to the control (Table 2). Reduced glutathione (GSH) was increased significantly (*p* < 0.01) in the liver and brain tissues of birds fed higher doses of ZnO-NPs (30 and 60 mg/kg diet) compared to the control (Table 2). The concentration of GSH in liver and brain tissue of birds fed lowest dose of ZnO-NPs (15 mg/kg diet) remained comparable to the control (Table 2). However, all studied doses of ZnO-NPs induced a significant reduction (*p* < 0.05) in oxidized glutathione (GSSG) concentrations in liver and brain tissue of Japanese quails compared to the control (Table 2).

**Table 2:** Effect of dietary ZnO-NPs supplementation on lipid peroxidation and oxidative stress biomarkers in liver and brain tissues of Japanese quails

**Parameters**	**Dietary groups**				**SEM**	***P* values**
	**Group 1**	**Group 2**	**Group 3**	**Group 4**		
**Liver**						
MDA(nM/g tissue)	27.20^a^	21.50^b^	19.63^b^	17.63^b^	1.39	0.04
NO (nM /g tissue)	0.15^a^	0.46^b^	0.52^b^	0.67^c^	0.06	0.0001
GSH (nM /g tissue)	12.50^a^	12.72^a^	17.95^b^	18.19^b^	0.89	0.002
GSSG (nM /g tissue)	0.29^a^	0.21^b^	0.20^b^	0.16^b^	0.01	0.04
**Brain**						
MDA (nM /g tissue)	23.48^a^	16.38^b^	14.77^b^	14.47^b^	1.33	0.02
NO (nM /g tissue)	0.16^a^	0.36^b^	0.41^b^	0.52^b^	0.04	0.006
GSH (nM /g tissue)	9.05^a^	11.32^a^	15.51^b^	16.44^b^	1.01	0.003
GSSG (nM /g tissue)	0.24^a^	0.16^b^	0.14^b^	0.12^b^	0.01	0.04

^a−c^ Means within a row not sharing a common superscript differ significantly with corresponding p value

Group 1: control; Group 2: ZnO-NPs 15 mg/kg diet; Group 3: ZnO-NPs 30 mg/kg diet; Group 4: ZnO-NPs 60 mg/kg diet

*Abbreviations*: *ZnO-NPs* zinc oxide nano particles, *nM* nanomole, *SEM* Standard error of mean, *MDA* malonaldehyde, *NO* Nitric oxide, *GSH* reduced glutathione, *GSSG* oxidized glutathione

### Effect of ZnO-NP supplementation on the gene expression of selected antioxidant enzymes and pro-inflammatory cytokines in liver and brain tissues

**Fig. 2:**
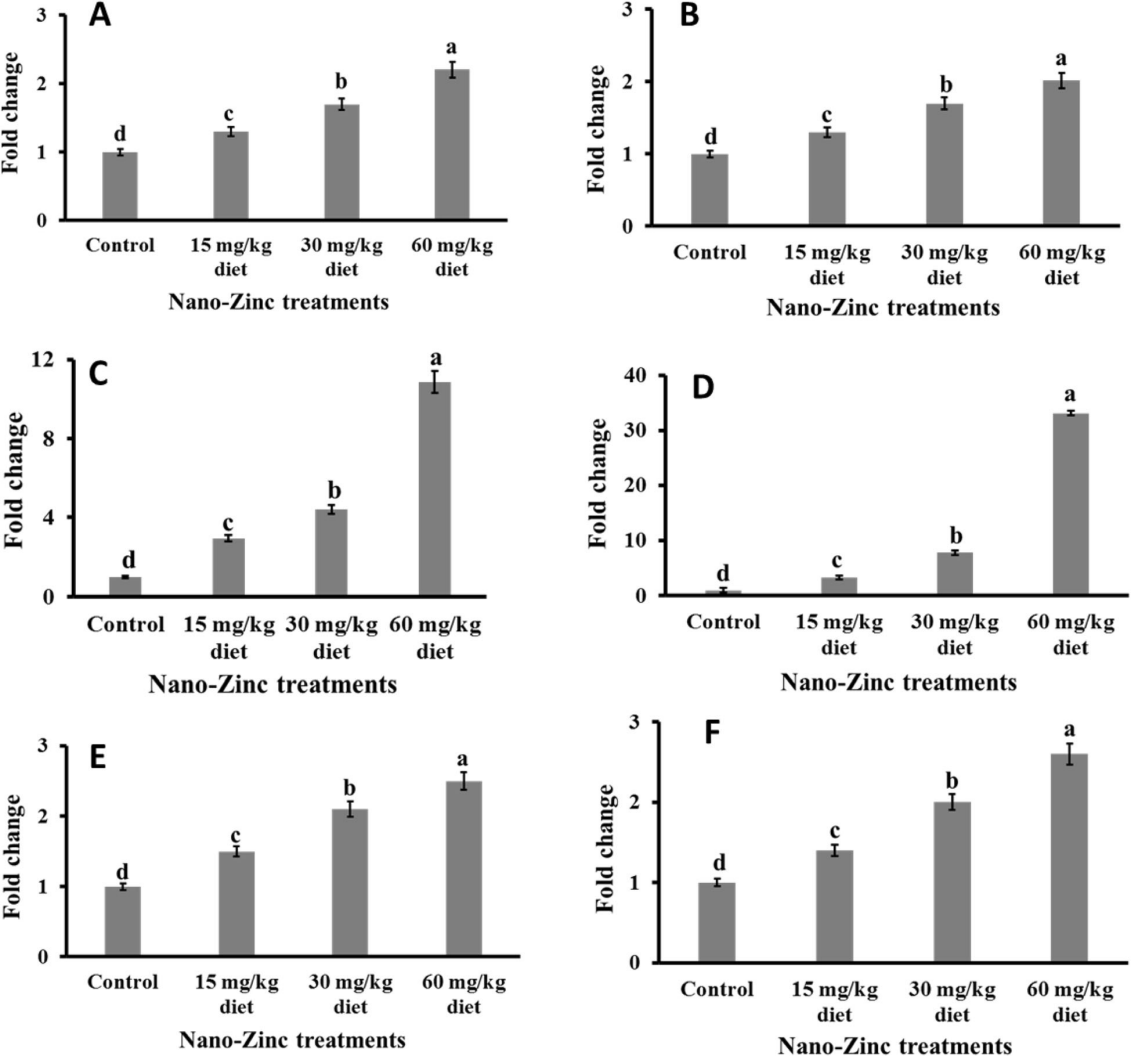
The differential expression of **a** Superoxide dismutase (SOD1), **b** Catalase (CAT), **c** Cellular Glutathione peroxidase 1 (GPX1), **d** Cellular Glutathione peroxidase 7 (GPX7), **e** interleukins (IL-6) and **f** interferon α (IFN-α) genes expressions in the liver of Japanese quail fed on diets supplemented with different levels of ZnO-NPs (15, 30, 60 mg/kg diet), β-actin gene was an internal reference gene. The data expressed as mean ± SEM

**Fig. 3:**
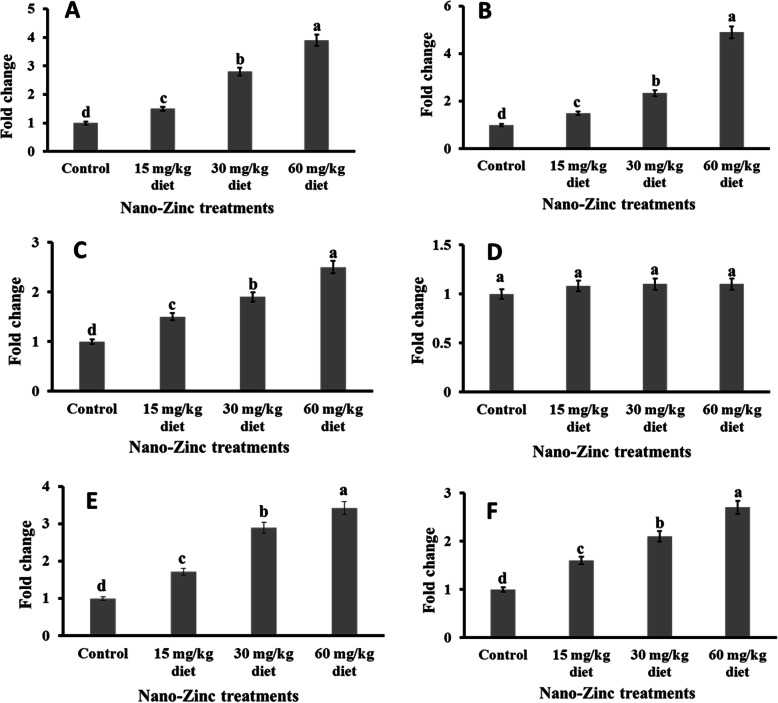
The differential expression of **a** Superoxide dismutase (SOD1), **b** Catalase (CAT), **c** Cellular Glutathione peroxidase 1 (GPX1), **d** Cellular Glutathione peroxidase 7 (GPX7), **e** interleukins (IL-6) and **f** interferon α (IFN-α) genes expressions in the brain tissue of Japanese quail fed on diets supplemented with different levels of ZnO-NPs (15, 30, 60 mg/kg diet), β-actin gene was an internal reference gene. The data expressed as mean ± SEM

In a dose-dependent manner, all studied doses of ZnO-NPs induced significant upregulation of the mRNA levels of the SOD-1 (Fig. 2a), CAT (Fig. 2b), GPX-1 (Fig. 2c), GPX-7 (Fig. 2d), IL-6 (Fig. 2e) and IFN-α (Fig. 2f) genes in the liver tissue of Japanese quails compared to the control. All studied doses of ZnO-NPs induced significant upregulation of the mRNA levels of the SOD-1 (Fig. 3a), CAT (Fig. 3b), GPX-1 (Fig. 3c), IL-6 (Fig. 3e) and IFN-α (Fig. 3f) genes in the brain tissue of Japanese quails compared to the control in a dose-dependent manner. However, the level of gene expression of GPX-7 remained unchanged in the brain tissue of Japanese quails fed ZnO-NPs compared to the control (Fig. 3d).

### Effect of dietary ZnO-NP supplementation on zinc concentrations in liver and brain tissues

Inclusion of ZnO-NPs in the diet of Japanese quails induced a significant increase in the zinc concentration in both liver and brain tissues compared to the control (Table 3). Liver tissue retained a higher zinc concentration than brain tissue (Table 3). The retention of zinc in either liver or brain tissue was dose independent (Table 3).

**Table 3:** Effect of dietary ZnO-NPs supplementation on zinc concnetrations in liver and brain tissues of Japanese quails

Tissues	Unit	Dietary groups				SEM	P values
		Group 1	Group 2	Group 3	Group 4		
Liver	PPb/g tissue	1.10^Aa^	5.20^Ab^	5.40^Ab^	5.15^Ab^	1.34	0.05
Brain	PPb/g tissue	1.09^Aa^	2.80^Bb^	2.89^Bb^	2.05^Bb^	1.07	0.04

^A−B^ Means within a column not sharing a common superscript differ significantly with corresponding *p* value

^a−c^ Means within a row not sharing a common superscript differ significantly with corresponding *p* value

Group 1: control; Group 2: ZnO-NPs 15 mg/kg diet; Group 3: ZnO-NPs 30 mg/kg diet; Group 4: ZnO-NPs 60 mg/kg diet

*Abbreviations*: *ZnO-NPs* zinc oxide nano particles, *SEM* Standard error of mean, *PPb/g tissue* part per billion per a gram of tissue

## Discussion

### Characterization of ZnO-NPs

In the current study, ZnO-NPs were produced by green biosynthesis, and the chemical stabilization of spherical ZnO-NPs by the acacia template as a natural, biodegradable polymer is proposed for the action of nucleation and crystal growth of ZnO-NP crystallites [13]. This process is preceded by an induction period of Zn cations and acacia precursor polymer in the solid state. Hazardous solvents or chemicals were not used in this preparation procedure [14]. The electrostatic linkages and coordination between zinc cations and hydrophilic functional hydroxyl groups of acacia were developed when zinc nitrate [(Zn(NO_3_)_2_] was added to the polymer under strong mechanical milling. Furthermore, the addition of NaOH billets converted the zinc cations coordinated by acacia into Zn(OH)_2_–acacia [15]. The obtained zinc hydroxide Zn(OH)_2_ is converted to ZnO-NP crystals via a further calcination process. The growth rate of ZnO-NP nuclei is controlled by the polymer, which governs the size distribution. The spherical shape of ZnO-NPs, as reflected by the HRTEM image, clearly indicated that each particle is very small in size and that the obtained size is approximately 40 nm. The FESEM images indicated that the ZnO-NPs are identical, spherical, have a smooth surface and have an approximate particle size of 60–95 nm. The average size was determined to be approximately 130 nm, which is higher than the value obtained from HRTEM and FESEM. This increase in size could be attributed to the swelling of the stabilizing coating polymer acacia when dispersed in deionized water. The apparent Zeta potential is approximately − 42 mV, which indicates relatively high repulsive forces present in the solution that in turn positively reflect the stability of the nanoparticles [14].

### Effect of dietary ZnO-NP supplementation on selected serum biochemical parameters

ZnO-NPs were safe with respect to liver function, as reflected by unchanged values of the activities of AST and ALT and concentrations of total proteins, albumin and globulin in the serum of Japanese quail fed all studied doses of ZnO-NPs compared to the control. This finding supported earlier results of ZnO-NPs in broilers [10] and weaned piglets [12]. Only the highest dose of ZnO-NPs (60 mg/kg diet) was able to induce a significant decrease in the concentration of TC in the serum of Japanese quail compared to other groups, including the control. It has been published that higher doses of ZnO-NPs (100 and 200 mg/kg diet) decreased TC concentrations effectively in the plasma of laying hens [16]. Only moderate (30 mg/kg) and high doses (60 mg/kg diet) of ZnO-NPs reduced TAG significantly in the serum of quails compared to the low dose and control. The reduction in TAG may be attributed to the reduction in lipid biosynthesis induced by ZnO-NPs, as indicated earlier in broilers [8] and in laying hens [16]. The hypolipidemic effect of ZnO-NPs may be attributed to the incorporation of zinc in the structure of metalloenzymes associated with lipid metabolism [17]. The highest dose of ZnO-NPs (60 mg/kg diet) was exclusively able to induce a significant increase in the concentration of zinc in the serum of Japanese quail compared to the other groups, including the control. Similar findings have been indicated earlier in broilers [18] and in laying hens [19].

### Effect of dietary ZnO-NP supplementation on lipid peroxidation, oxidative stress biomarkers and gene expression of selected antioxidant enzymes and pro-inflammatory cytokines in liver and brain tissues

The current findings indicated a significant reduction in MDA concentrations in liver and brain tissues of birds fed all studied doses of ZnO-NPs compared to the control. Because MDA is a well-known biomarker of lipid peroxidation [20], the current findings confirmed the antioxidant effect of ZnO-NPs in quail tissues at all studied doses, as demonstrated earlier in other birds [9, 19, 21]. Of course, zinc plays the main role in this antioxidant effect [22]. The antioxidant effects of ZnO-NPs in quail [37] and birds [23–25] were supported by the current results, which reflected a significant increase in NO in the liver and brain tissues of Japanese quails fed ZnO-NPs compared to the control. NO modifies cellular processes that protect cells and tissue from oxidative damage. In addition to the antioxidant chemistry, NO protects against cell death mediated by H_2_O_2_, alkylhydroperoxides, and xanthine oxidase. The attenuation of metal/peroxide oxidative chemistry, as well as lipid peroxidation, appears to be the major chemical mechanisms by which NO may limit oxidative injury [38]. Both level of 120 and 200 mg Zn/kg diet increased the sum of serum nitrite and nitrate in broilers [39]. Usually, most glutathione is in reduced form (GSH), but in the presence of free radicals, it is converted to glutathione disulfide (GSSG) at a fast rate [26]. New evidence of the antioxidant effect of dietary ZnO-NPs in quail liver and brain has been submitted in the current study when all doses, particularly the higher doses of ZnO-NPs induced a significant increase in GSH accompanied by a significant decrease in GSSG in relevant tissues. The positive correlation between zinc and GSH has been reported earlier [26–29]. The antioxidant effect of ZnO-NPs in liver and brain tissues of Japanese quails has been confirmed at the molecular level as reflected by the significant upregulation of antioxidant enzyme (SOD-1, CAT, and GPX-1) genes. The significant upregulation of SOD-1 because of dietary supplementation of ZnO-NPs in liver and brain tissues of Japanese quails can be explained by the potential structural role of zinc as a co-factor of superoxide dismutase [30]. Oxidative stress induced upregulation of SOD-1 gene expression, which is usually followed by upregulation of the expression of CAT and GPX genes [31]. The significant upregulation of antioxidant enzymes because of ZnO-NP supplementation has been confirmed in broilers [9, 32] and tilapia fish [11]. The current study indicated that GPX-7 gene expression was detected in liver only and not in brain tissue. The highest GPX activities were observed in the liver, kidney, heart, lung and yolk sac membrane, whereas the lowest activities were observed in the muscle and brain of broiler chickens [33]. The specific activity of the enzyme in the liver was 6.1 times greater than that in the brain in chicken [34]. This might also be the case in Japanese quail, which requires further investigation. The anti-inflammatory and antiviral effects of ZnO-NPs in liver and brain tissues of Japanese quails have been observed at the molecular level, as reflected by the significant upregulation of cytokine (IFN-α and IL-6) genes. This effect has been demonstrated earlier in vitro [35] and in humans [36]. In addition, the well-known role of Zinc on activation of monocyte and release of IFN-α and IL-6 has been documented earlier in human [35, 36].

### Effect of dietary ZnO-NP supplementation on zinc concentrations in liver and brain tissues

The significant increase in zinc concentration in both liver and brain tissues of ZnO-NP-treated Japanese quails compared to the control confirmed that tissue mineral biodistribution can be used as an index of mineral storage in the body [37]. Previous works indicated that Zn levels in the liver and other tissues were significantly increased by dietary inclusion of Zn either in the form of zinc oxide [40] or nano-zinc oxide [41] in broiler chickens. The retention of zinc in either liver or brain tissue was dose independent. In the current study, dietary ZnO-NPs induced a significant increase in the zinc concentration in both liver and brain tissues of Japanese quails compared to the control. This observation is supported by the same finding that was reported recently in broiler chickens [41]. In the current study, liver tissue retained a higher zinc concentration than that of brain tissue. This observation is supported by the accumulation of Zn in the liver of chickens supplemented with graded levels of Zn sulfate [42]. The liver is an organ in the body that processes blood and helps to remove unwanted substances [42]. Zinc in the form of nanoparticles has the ability to penetrate hepatic cells via the blood or interstitial space better than the brain and brain barriers [41].

## Conclusions 

The current study concluded that the inclusion of ZnO-NPs (30 and 60 mg/kg) induced a significant decrease in serum TAG and was safe for liver function. In general, the inclusion of ZnO-NPs reduced the MDA and upregulated the mRNA levels of antioxidant enzymes (SOD1, CAT and GPX1) and pro-inflammatory cytokines (IFN-α and IL-6) in liver and brain tissues in a dose-dependent manner. Therefore, the current study recommended the inclusion of the studied dose of ZnO-NPs, particularly 60 mg/kg, in the diet of Japanese quails to improve antioxidant and immune status. Future studies are needed to explain the reason behind the expression of GPX-7 genes in the liver only and not in brain tissue of Japanese quails.

## Methods

### Preparation of ZnO-NPs

ZnO-NPs were prepared in the Textile Research Division, National Research Centre, Dokki, Egypt, by the wet chemical method using Acacia (Merck). Briefly, 0.4 g of Acacia was mixed with 0.9 g of zinc nitrate hexahydrate (Zn(NO_3_)_2_.6H_2_O; Sigma-Aldrich) in the presence of 0.025 g of sodium hydroxide (Fisher Scientific). The mixture was stirred and ground vigorously using a ball mill mixer for 10 min. The mixture was then washed with deionized water to separate the product, Zn(OH)_2_, and to remove the unreacted reagents. The product was air dried at 80 °C and then calcinated at 800 °C for 1 h to obtain pure ZnO-NPs.

### Characterization of ZnO-NPs

The particle size and size distribution of the obtained ZnO-NPs were investigated by using a high-resolution transmission electron microscope (HRTEM; JEOL-JEM-1200) [13]. The HRTEM samples were prepared by dropping a dilute suspension of ZnO-NPs onto copper-coated grids. The morphological features of the synthesized ZnO-NPs were examined by field emission scanning electron microscopy (FESEM; Quanta FEG 250) with the field emission technique [13]. The FESEM images of ZnO-NPs were taken at a magnification of 12,000X. Energy dispersive X-ray spectra (EDS) were connected to FESEM to determine the existence of zinc. The size distribution and the surface charge of the ZnO-NP preparations were investigated by using a Marvelan Zetasizer-SE [13].

### Study collaboration

The current study is a collaborative project between King Faisal University, Saudi Arabia and Benha University, Egypt. This project was funded by the Deanship of Scientific Research, King Faisal University, Saudi Arabia (Grant # 186,202). The Deanship of Scientific Research, King Faisal University, Saudi Arabia, approved the design of the study and provided the total funding of the study, whereas the experimental procedures and management conditions used in this study were carried out in accordance with the IUCN policy statement on research involving species at risk of extinction and approved by the Animal Care and Use Committee of Benha University, Faculty of Veterinary Medicine, Egypt (BUFVTM; permission # 03-10-2018).

### Birds and experimental design

Eighty Japanese quails (*Coturnix coturnix japonica*; 45 days old) were provided by the Quails Unit, Center of Agriculture Production Technology, College of Agriculture, Cairo University, Egypt. After arrival, quails were housed in 16 wire cages equipped with trough feeders and automatic drinkers at the farm of the Faculty of Agriculture, Benha University, Egypt. Birds were randomly divided into four treatments (20 birds for each) with 4 replicates (5 birds each). Birds of the first group were fed basal diet alone and served as a control. Birds of groups 2–4 were fed a basal diet supplemented with ZnO-NPs at doses of 15 mg/kg, 30 mg/kg and 60 mg/kg for 60 days. The basal diet was formulated according to the National Research Council (NRC) [43]. The composition of the basal diet is listed in Table 4. The premix used in the diet did not contain additional zinc. All diets within a period had the same chemical composition. Birds had free-choice access to diets and clean water (i.e., *ad libitum*) in an entirely randomized design during the experimental period (60 days). The basal diet was analysed for zinc content by using an AA-6800 model flame atomic absorption spectrophotometer [44].

**Table 4:** Diet composition and calculated chemical analysis

Ingredients	Level %
Yellow corn grains	57.1
Soybean meal 44%	28.47
Corn gluten meal	4.5
Vegetable oil	1.8
Limestone	5.55
Dicalcium phosphate	1.73
Sodium chloride	0.33
Vitamin and mineral premix^1^	0.28
L-lysine	0.1
DL-methionine	0.14
**Nutrient specifications**	
ME MJ/kg	12.14
CP (%)	20
Ca (%)	2.5
Available P (%)	0.4
Calculated zinc (mg/kg)	60.44 [41]
Analyzed zinc (mg/kg)	66.6 ± 1.5 (group 1); 73.0 ± 1.3 (group 2); 81.4 ± 1.2 (group 3); 84.9 ± 1.1 (group 4)
Methionine (%)	0.47
Lysine (%)	1.01

^1^Vitamin and mineral premix supplied each kg of feeds with: Vitamin A 12,000 IU; vitamin D_3_ 2000 IU; vitamin E 10 mg; vitamin K_3_ 2 mg; vitamin B_1_ 1 mg; vitamin B_2_ 5 mg; vitamin B_6_ 1.5 mg; vitamin B_12_ 0.01 mg; Biotin 0.05 mg; pantothenic acid 10 mg; Nicotinic acid 30 mg; Folic acid 1 mg; Manganese 60 mg; Iron 30 mg; Copper 10 mg; Iodine 1 mg; Selenium 0.01 mg; Cobalt 0.01 mg. The zinc content of the premix was13600 mg/kg

### Sample collection and biochemical analysis of oxidative stress biomarkers

At the end of the experiment, all birds were euthanised by sodium pentobarbital (100 mg/kg) infused intravenously into the wing vein [45]. Afterwards, blood was collected in a plain vacutainer, whereas tissues (liver and brain) were stored frozen at -80 °C. The collected blood samples were centrifuged at 1008 *g* for 10 min, and the obtained sera were stored frozen at -20 °C until the time of analysis of the activities of alanine aminotransferase (ALT) and aspartate aminotransferase (AST) [46], total cholesterol (TC; [47]), triacylglycerol (TAG; [48]), total proteins and albumin by using commercially available kits (Spinreact, Spain) with Catalogue Numbers TK41274, MD41264, SP41021, 41,030, MD1001291 and MX1001020, respectively. After thawing, the liver and one portion of the brain tissue were homogenized in ice-cold phosphate buffer saline (pH 7.4) to prepare 10% (w/v) homogenate and used for estimation of malondialdehyde (MDA; [49]), nitric oxide (NO; [50]), reduced glutathione (GSH), and oxidized glutathione (GSSG) concentrations [51] by HPLC. In addition, serum and tissue homogenates were used for the determination of zinc (Zn) by using an AA-6800 model flame atomic absorption spectrophotometer [44].

### Gene expression analysis of selected antioxidant enzymes and pro-inflammatory cytokines

Liver and brain tissue samples were ground by a Tissue Lyser LT apparatus (QIAGEN). Total RNA was extracted from the suspension of cells using the SV Total RNA Isolation System (Promega Cat. # Z3100). The reverse transcriptase reaction of RNA was conducted by using the QuantiTect® Reverse Transcription Kit (Qiagen, Cat. # 205,311). Triplicate PCRs were performed for each complementary DNA (cDNA) sample in addition to the non-template control (NTC) and negative cDNA template. Oligonucleotide names, sequences and accession numbers of qRT-PCR primers are presented in Table 5. Primers for superoxide dismutase-1 (SOD1; [52]), glutathione peroxidase-1 (Gpx1; [53]), glutathione peroxidase-7 (Gpx7; [52]), interleukin-6 (IL-6; [54]), interferon alpha (IFN-α; [55]) and beta actin (ß-actin; [56]) were selected from the literature. However, the primer for the catalase (CAT) gene was designed using Primer 3 (http://www.ncbi.nlm.nih.gov/tools/primer-blast/; NC_029520.1). Each PCR reaction was composed of 2.5 µl of cDNA, 12.5 µl of SYBR Green Mix (Qiagen Cat. No. 204,143), 0.3 µM of each forward and reverse primer, and 1 µl of RNase inhibitor, and the final volume was adjusted to 25 µl by adding RNase-Free water. Reactions were assessed on an AriaMx Real-Time PCR System (Agilent Technologies), two-step cycling protocol, under the following conditions: 95 °C for 10 min and 40 cycles of 95 °C for 15 s followed by 60 °C for 60 s. The expression levels were normalized to those of β-actin as an internal reference gene. Changes in the expression of the studied genes are presented as n-fold changes relative to the corresponding controls. Relative gene expression ratios (RQ) were estimated using the formula: RQ = 2^−ΔΔCT^ [57].

**Table 5:** Oligonucleotide name, sequence and accession numbers of qRT-PCR primers

Gene	Sequence	Accession #	References
SOD1	F: TGGACCTCGTTTAGCTTGTG	XM_015881247.1	[50]
	R: ACACGGAAGAGCAAGTACAG		
CAT	F: CCTGACTATGGTGCGCGTAT	NC_029520.1	Designed
	R: CAGACACACGAGAAGTGGCT		
GPX1	F: CAG TTC GGG CAT CAG GAGAA	AB371709.1	[51]
	R: CGA GGA ACT TGC TCGAAA GTT ACC AGG		
GPX7	F: TTGTAAACATCAGGGGCAAA	XM_015870585.1	[50]
	R: TGGGCCAAGATCTTTCTGTAA		
IL-6	F: CAACCTCAACCTGCCCAA	XM_015853679.1	[52]
	R: GGAGAGCTTCCTCAGGCATT		
IFN-α	F: CCTTGCTCCTTCAACGACA	AB154298.1	[53]
	R: CGCTGAGGATACTGAAGAGGT		
β-actin	F: CTGGCACCTAGCACAATGAA	XM_015876619.1	[54]
	R: CTGCTTGCTGATCCACATCT		

*Abbreviations*: *qRT-PCR* quantitative real time polymerase chain reaction, *SOD1* super oxide dismutase-1, *CAT* catalase, *GPX1* glutathione peroxidase-1, *GPX7* glutathione peroxidase-7, *IL-6* interleukin 6, *IFN-α* interfron α, *β-actin* beta actin

### Statistical analysis

The collected data were analysed by multivariate analysis (factorial analysis). SPSS (version 16; IBM, Chicago, IL, USA) software was used. Duncan’s multiple comparison tests [58] were applied to compare the differences between dietary groups. The comparison between zinc concentrations in liver and brain tissues of each group conducted via independent samples t test. The independent factor was the tissue itself when comparing zinc concentration among group. However, the independent factor was the group itself when comparing zinc concentration in liver and brain tissues. The sample size calculation was performed using power and sample size program (www.power-analysis.com); the type 1 error was 0.05, and the power was 85%. Eighty birds were classified into 4 groups (20 birds for each). Each group allocated into 4 replicates (5 birds for each) as 4 × 4 × 5 factorial randomised controlled study design. This factorial randomised controlled study design reduced the number of birds used. The assessors were blinded to any stages of the methodological process.

## Data Availability

The datasets used and/or analyzed during the current study are available from the corresponding author on reasonable request.

## Abbreviations

ZnO-NPs: Zinc oxide Nano particles
FESEM: Field emission scanning electron microscopy
SOD1: Super oxide dismutase-1
CAT: Catalase
GPX1: Glutathione peroxidase-1
GPX7: Glutathione peroxidase-7
IL-6: Interleukin 6
IFN-α: Interfron α
β-actin: Beta actin
SEM: Standard error of mean
ALT: Alanine aminotransferase
AST: Aspartate aminotransferase
TC: Total cholesterol
TAG: Triacylglycerol
MDA: Malonaldehyde
NO: Nitric oxide
GSH: Reduced glutathione
GSSG: Oxidized glutathione
qRT-PCR: Quantitative real time polymerase chain reaction
PPb: Part per billion
nM: Nanomole

## References

[1] Zhang TY, Liu JL, Zhang JL, Zhang N, Yang X, Qu HX, Xi L, Han JC (2018). Effects of dietary zinc levels on the growth performance, organ zinc content, and zinc retention in broiler chickens. Braz J Poult Sci.

[2] Bratz K, Golz G, Riedel C, Janczyk P, Nockler K, Alter T (2013). Inhibitory effect of high-dosage zinc oxide dietary supplementation on Campylobacter coli excretion in weaned piglets. J Appl Microbiol.

[3] Aksu T, Aksu MI, Yoruk MA, Karaoglu M (2011). Effects of organically-complexed minerals on meat quality in chickens. Br Poult Sci.

[4] Suttle NF (2010). Mineral nutrition of livestock.

[5] Sekhon BS (2014). Nanotechnology in agri-food production: an overview. Nanotechnol Sci Appl.

[6] Tsai YH, Mao SY, Li MZ, Huang JT, Lien TF (2016). Effects of nanosize zinc oxide on zinc retention, eggshell quality, immune response and serum parameters of aged laying hens. Anim Feed Sci Techn.

[7] Song S, Qin Y, He Y, Huang Q, Fan C, Chen HY (2010). Functional nanoprobes for ultrasensitive detection of biomolecules. Chem Soc Rev.

[8] Ibrahim D, Ali HA, El-Mandrawy SA (2017). Effects of different zinc sources on performance, bio distribution of minerals and expression of genes related to metabolism of broiler chickens. Zagazig Vet J.

[9] Zhao CY, Tan SX, Xiao XY, Qiu XS, Pan JQ, Tang ZX (2014). Effects of dietary zinc oxide nanoparticles on growth performance and antioxidative status in broilers. Biol Trace Elem Res.

[10] Ahmadi F, Ebrahimnezjad Y, Ghalehkandi J, Sis N. The effect of dietary zinc oxide nanoparticles on the antioxidant state and serum enzymes activity in broiler chickens during starter. International Conference on Biological, Civil and Environmental Engineering. Dubai; 2014;26–28. doi:10.15242/iicbe.c0314120.

[11] Saddick S, Afifi M, Zinada OAA (2017). Effect of Zinc nanoparticles on oxidative stress-related genes and antioxidant enzymes activity in the brain of Oreochromis niloticus and Tilapia zillii. Saudi J Biol Sci.

[12] Wang C, Zhang L, Ying Z, He J, Zhou L, Zhang L, Zhong X, Wang T (2018). Effects of Dietary Zinc Oxide Nanoparticles on Growth, Diarrhea, Mineral Deposition, Intestinal Morphology, and Barrier of Weaned Piglets. Biol Trace Elem Res.

[13] Hussein J, El-Banna M, AbdelRazik T, El-Naggar ME (2018). Biocompatible zinc oxide nanocrystals stabilized via hydroxyethyl cellulose for mitigation of diabetic complications. Int J Biol Macromol.

[14] Hebeish A, Shaheen TI, El-Naggar ME (2016). Solid state synthesis of starch-capped silver nanoparticles. Int J Biol macromol.

[15] Shaheen TI, El-Naggar ME, Abdelgawad AM, Hebeish A (2016). Durable antibacterial and UV protections of in situ synthesized zinc oxide nanoparticles onto cotton fabrics. Int J Biol Macromol.

[16] Zhao Y, Li L, Zhang PF, Liu XQ, Zhang WD, Ding ZP, Wang SW, Shen W, Min LJ, Hao ZH (2016). Regulation of egg quality and lipids metabolism by Zinc Oxide Nanoparticles. Poult Sci.

[17] Al-Daraji HJ, Amen MH (2011). Effect of dietary zinc on certain blood traits of broiler breeder chickens. Int J Poult Sci.

[18] Bao Y, Choct M, Iji P, Bruerton K (2009). Optimal dietary inclusion of organically complexed zinc for broiler chickens. Br Poult Sci.

[19] Abedini M, Shariatmadari F, Karimi Torshizi M, Ahmadi H (2018). Effects of zinc oxide nanoparticles on the egg quality, immune response, zinc retention, and blood parameters of laying hens in the late phase of production. J Anim Physiol Anim Nutr.

[20] Nielsen F, Mikkelsen BB, Nielsen JB, Andersen HR, Grandjean P (1997). Plasma malondialdehyde as biomarker for oxidative stress: reference interval and effects of life-style factors. Clin Chem.

[21] Atakisi O, Atakisi E, Kart A (2009). Effects of dietary zinc and L-arginine supplementation on total antioxidants capacity, lipid peroxidation, nitric oxide, egg weight, and blood biochemical values in Japanase quails. Biol Trace Elem Res.

[22] Wasowicz W, Reszka E, Gromadzinska J, Rydzynski K (2003). The role of essential elements in oxidative stress. Comm Toxicol.

[23] Wink DA, Miranda KM, Espey MG, Pluta RM, Hewett SJ, Colton C, Vitek M, Feelisch M, Grisham MB (2001). Mechanisms of the antioxidant effects of nitric oxide. Antioxid Red Sig.

[24] Manwar SJ, Moudgal R, Sastry K, Mohan J, Tyagi J, Raina R (2006). Role of nitric oxide in ovarian follicular development and egg production in Japanese quail (Coturnix coturnix japonica). Theriogenology.

[25] Gutierrez FR, Mineo TW, Pavanelli WR, Guedes PM, Silva JS (2009). The effects of nitric oxide on the immune system during Trypanosoma cruzi infection. Mem Inst Oswaldo Cruz.

[26] Pinto E, Sigaud-kutner TC, Leitao MA, Okamoto OK, Morse D (2003). Colepicolo P Heavy metal–induced oxidative stress in algae. J Phycol.

[27] Powell SR (2000). The antioxidant properties of zinc. J Nutr.

[28] Nordmann R (1994). Alcohol and antioxidant systems. Alcohol Alcoholism.

[29] Ozturk A, Baltaci AK, Mogulkoc R, Oztekin E, Sivrikaya A, Kurtoglu E, Kul A (2003). Effects of zinc deficiency and supplementation on malondialdehyde and glutathione levels in blood and tissues of rats performing swimming exercise. Biol Trace Elem Res.

[30] Yuan J, Xu Z, Huang C, Zhou S, Guo Y (2011). Effect of dietary Mintrex-Zn/Mn on performance, gene expression of Zn transfer proteins, activities of Zn/Mn related enzymes and fecal mineral excretion in broiler chickens. Anim Feed Sci Technol.

[31] Ahmad H, Tian J, Wang J, Khan MA, Wang Y, Zhang L, Wang T (2012). Effects of dietary sodium selenite and selenium yeast on antioxidant enzyme activities and oxidative stability of chicken breast meat. J Agri Food Chem.

[32] Hafez A, Nassef E, Fahmy M, Elsabagh M, Bakr A, Hegazi E (2019). Impact of dietary nano-zinc oxide on immune response and antioxidant defense of broiler chickens. Environ Sci Pollut Res.

[33] Surai PF, Speake BK, Noble RC, Sparks NH (1999). Tissue-specific antioxidant profiles and susceptibility to lipid peroxidation of the newly hatched chick. Biol Trace Elem Res.

[34] Surai P, Kochish I, Fisinin V (2018). Glutathione peroxidases in poultry biology: Part 1. Classification and mechanisms of action. Worlds Poult Sci J.

[35] Cakman I, Kirchner H, Rink L (1997). Zinc supplementation reconstitutes the production of interferon-alpha by leukocytes from elderly persons. J Interferon Cytokine Res.

[36] Berg K, Bolt G, Andersen H, Owen TC (2001). Zinc potentiates the antiviral action of human IFN-α tenfold. J Interferon Cytokine Res.

[37] Wedekind KJ, Hortin AE, Baker DH (1992). Methodology for assessing zinc bioavailability: efficacy estimates for zincmethionine, zinc sulfate, and zinc oxide. J Anim Sci.

[38] Wink DA, Miranda KM, Espey MG, Pluta RM, Hewett SJ, Colton C, Vitek M, Feelisch M, MB Grisham (2001). Mechanisms of the antioxidant effects of nitric oxide. Antioxid Redox Signal.

[39] Sajadifar S, Miranzadeh H, Moazeni M (2013). Effect of zinc on humoral and cell-mediated immunity of broilers vaccinated against coccidiosis. Iranian J Parasitol.

[40] Kakhki AMR, Bakhshalinejad R, Hassanabadi A, Ferket P (2017). Effects of dietary organic zinc and α-tocopheryl acetate supplements on growth performance, meat quality, tissues minerals, and α-tocopherol deposition in broiler chickens. Poult Sci.

[41] Ramiah SK, Awad EA, Mookiah S, Idrus Z (2019). Effects of zinc oxide nanoparticles on growth performance and concentrations of malondialdehyde, zinc in tissues, and corticosterone in broiler chickens under heat stress conditions. Poult Sci.

[42] Gundoğdu A, Yardim O, Bat L, Culha Türk S (2009). Accumulation of zinc in liver and muscle tissues of rainbow trout (Onchorhyncus mykiss Walbaum 1792). Fresen Environ Bull.

[43] National Research Council (NRC). Nutrient requirements of poultry. 9th. Washington, DC. The National Academies Press; 1994.

[44] El-Bahr SM, Abdelghany AM (2015). Heavy metal and trace element contents in edible muscle of three commercial fish species, and assessment of possible risks associated with their human consumption in Saudi Arabia. J Adv Vet Anim Res.

[45] Magubane MM, Lembede BW, Erlwanger KH, Chivandi E, Donaldson J. Fat absorption and deposition in Japanese quail (Coturnix coturnix japonica) fed a high fat diet. J South Afr Vet Assoc. 2013;84(1), Art. #384, 7 pages. 10.4102/jsava.v84i1.384. 10.4102/jsava.v84i1.384 23718824

[46] Reitman S, Frankel S (1957). A colorimetric method for the determination of serum glutamic oxalacetic and glutamic pyruvic transaminases. Am J Clin Pathol.

[47] Wieland H, Seidel D (1983). A simple specific method for precipitation of low density lipoproteins. J Lipid Res.

[48] Fossati P, Prencipe L (1982). Serum triglycerides determined colorimetrically with an enzyme that produces hydrogen peroxide. Clin Chem.

[49] Karatepe M (2004). Simultaneous determination of ascorbic acid and free malondialdehyde in human serum by HPLC/UV. LC-GC North America.

[50] Papadoyannis IN, Samanidou VF, Nitsos CC (1999). Simultaneous determination of nitrite and nitrate in drinking water and human serum by high performance anion-exchange chromatography and uv detection. J Liquid Chromat Related Technol.

[51] Jayatilleke E, Shaw S (1993). A high-performance liquid chromatographic assay for reduced and oxidized glutathione in biological samples. Anal Biochem.

[52] Bastos MS, Del Vesco AP, Santana TP, Santos TS, de Oliveira Junior GM, Fernandes RPM, Barbosa LT, Gasparino E (2017). The role of cinnamon as a modulator of the expression of genes related to antioxidant activity and lipid metabolism of laying quails. PloS One.

[53] Wilaison S, Mori M (2009). Cloning and expression of cellular glutathione peroxidase (GPX1) in Japanese quail (Coturnix japonica). J Poult Sci.

[54] Vinkler M, Svobodová J, Gabrielová B, Bainová H, Bryjová A (2014). Cytokine expression in phytohaemagglutinin-induced skin inflammation in a galliform bird. J Avian Biol.

[55] Uno Y, Usui T, Fujimoto Y, Ito T, Yamaguchi T (2012). Quantification of interferon, interleukin, and Toll-like receptor 7 mRNA in quail splenocytes using real-time PCR. Poult Sci.

[56] Wang D, Xu C, Wang T, Li H, Li Y, Ren J, Tian Y, Li Z, Jiao Y, Kang X (2016). Discovery and functional characterization of leptin and its receptors in Japanese quail (Coturnix japonica). Gen Comp Endocrinol.

[57] Livak KJ, Schmittgen TD (2001). Analysis of relative gene expression data using real-time quantitative PCR and the 2 – ∆∆CT method. Methods.

[58] Duncan DB (1955). Multiple range and multiple F-test. Biometrics.

